# Design and Application of Hetero-Multicomponent Metal Oxide Photocatalysts for Wastewater Treatment: Ti–Cu–Zn Catalysts and Future Research Directions

**DOI:** 10.3390/molecules31020299

**Published:** 2026-01-14

**Authors:** Maria-Anthoniette Oghenetejiro Onoriode-Afunezie, Justinas Krutkevičius, Agnė Šulčiūtė

**Affiliations:** Department of Physical and Inorganic Chemistry, Faculty of Chemical Technology, Kaunas University of Technology, Radvilėnu pl. 19, 50254 Kaunas, Lithuania; justinas.krutkevicius@ktu.edu (J.K.); agne.sulciute@ktu.lt (A.Š.)

**Keywords:** photocatalysis, advanced oxidation processes, environmental nanotechnology, wastewater treatment, ternary heterostructures, agricultural pollutants

## Abstract

Hetero-multicomponent metal oxide catalysts are attracting increasing attention for wastewater remediation due to their tunable band structures, synergistic redox activity, and enhanced stability. This review thoroughly evaluates recent progress in the synthesis and application of such catalysts, highlighting Ti–Cu–Zn nanostructures as a representative case study. We examine synthesis approaches—including hydrothermal, biosynthesis, precipitation, and spray-based methods, with additional insight into sol–gel and other less commonly applied techniques—with emphasis on their suitability for constructing layered and multicomponent heterostructures. Mechanistic aspects of photocatalysis, Fenton and Fenton-like processes, adsorption, and electrochemical routes are discussed, with particular focus on charge separation, reactive oxygen species (ROS) generation, and pollutant-specific degradation pathways. Comparative performance metrics against antibiotics, pesticides, dyes, and fertilizers are analyzed, alongside considerations of leaching, reusability, and scale-up potential. Importantly, while significant progress has been made for organic micropollutants, applications in heavy metal remediation remain scarce, highlighting an urgent research gap. By situating Ti–Cu–Zn systems within the broader class of multicomponent catalysts, this review not only synthesizes current advances but also identifies opportunities to expand their role in sustainable wastewater management, including field deployment, regulatory compliance, and integration into decentralized treatment systems.

## 1. Introduction

The intensification of agricultural activities over recent decades has contributed significantly to global food security, but at the cost of increasing environmental degradation [1,2]. One of the most critical issues is the generation of agricultural wastewater. Agricultural wastewater is a significant environmental concern due to its contamination with various pollutants that adversely affect water quality and ecosystems. The runoff from agricultural activities introduces nutrients [3], pathogens [4], and chemicals into water bodies [4,5], causing detrimental effects on both human health and aquatic life. Increased nutrient levels lead to algal blooms, which reduce light penetration and harm aquatic habitats [4]. Contaminants can alter soil chemistry, affecting crop growth and soil biodiversity [5], and contaminated water sources can lead to an increased incidence of diseases, affecting community health and agricultural productivity [4]. Agricultural wastewater treatment faces significant challenges with both conventional methods and emerging nanomaterial-based technologies. While conventional treatments such as sedimentation, activated sludge, and membrane filtration are often insufficient to completely degrade or remove emerging pollutants, they also often struggle with inefficiency [6], environmental impact [7] and high costs [8].

Nanomaterials offer innovative solutions as there is a growing interest in advanced oxidation processes (AOPs) [9], particularly photocatalysis, due to their ability to mineralize a broad spectrum of organic compounds [10] under mild conditions [9] for the degradation of such refractory pollutants [9,10]. Heterogeneous photocatalysis has emerged as a promising technology for environmental remediation, particularly in the degradation of organic contaminants [11]. This process uses light energy to generate reactive oxygen species (ROS), which are capable of breaking down a wide range of organic pollutants [12]. The technology primarily uses semiconductor materials, which are activated by light to produce electron–hole pairs that interact with surface molecules, leading to the formation of ROS [13]. These ROS are highly effective in the oxidization and decomposition of organic contaminants, making heterogeneous photocatalysis a versatile and efficient solution for environmental challenges [13]. Among photocatalysts, titanium dioxide (TiO_2_) [14] is notable for its stability, abundance, and photocatalytic performance [14] under ultraviolet (UV) light. However, the rapid recombination of photogenerated charge carriers and their wide band gap (~3.2 eV) significantly limit their visible-light activity [15] and overall efficiency in practical applications [16]. To address these limitations, researchers have increasingly explored heterostructured and multicomponent nanomaterials [17], which offer synergistic benefits through enhanced light absorption, improved charge separation, and tailored surface chemistry.

In particular, sandwiched nanostructures—layered systems composed of two or more metal oxides or semiconductors—have emerged as promising architectures for optimizing photocatalytic performance. These structures improve photocatalytic efficiency by improving light absorption [18], charge separation, and surface area [19], which are critical for applications such as environmental remediation and energy conversion. The integration of different materials in a layered manner allows for the tuning of electronic properties and the extension of light absorption into the visible spectrum [18], thus enhancing the overall photocatalytic activity [19]. These structures can be designed to facilitate directional charge transfer and offer tunable interfaces that improve redox activity and durability.

In this context, metal-modified TiO_2_ systems, especially those incorporating copper (Cu) and zinc (Zn), have attracted considerable attention [20,21]. Cu-based materials (e.g., CuO, Cu_2_O) act as visible light absorbers and electron sinks [22,23], thus reducing recombination rates [24]. On the other hand, Zn species (e.g., ZnO or Zn^2+^ dopants) can enhance surface reactivity [25] and impart antimicrobial functionality [26]; an important feature for the treatment of wastewater with high microbial loads. The combination of titanium, copper, and zinc in a sandwiched nanostructure has demonstrated synergistic effects in photocatalytic applications [21].

Although hetero-multicomponent metal oxide catalysts can be constructed from a wide range of metal combinations, this review adopts TiO_2_–CuO–ZnO (known as Ti–Cu–Zn) systems as a representative and instructive platform. This choice is motivated by two reasons. The first is that TiO_2_, CuO/Cu_2_O, and ZnO represent three of the most extensively studied and industrially relevant oxide semiconductors in water treatment and secondly, the complementary electronic properties of TiO_2_, CuO, and ZnO, which enable diverse heterojunction configurations, including type-II and p–n junctions, as well as effective charge separation through interfacial band alignment. In addition, Ti–Cu–Zn materials have been investigated across multiple advanced oxidation pathways, including photocatalysis, Fenton-like reactions, and antimicrobial processes, allowing structure–property–function relationships to be systematically compared. Importantly, the Ti–Cu–Zn system integrates earth-abundant, relatively low-toxicity metal oxides with established synthesis scalability, making it a suitable model system for assessing both mechanistic performance and sustainability considerations. While other multicomponent oxide combinations are briefly discussed where relevant, Ti–Cu–Zn catalysts are emphasized to provide a coherent and focused framework for critical analysis.

Advanced oxidation processes (AOPs) encompass a diverse set of chemical, photochemical, and electrochemical technologies, including ozonation, Fenton and Fenton-like reactions, persulfate activation, and photocatalysis. While these methods have demonstrated effectiveness for wastewater treatment, this review deliberately focuses on photocatalytic AOPs due to their unique advantages, including tunable band structures, controlled reactive oxygen species (ROS) generation, potential for solar-driven operation, and reduced reliance on continuous chemical inputs. An overview of major AOPs and their respective strengths and limitations is provided in Section 2 to contextualize this focus and highlight why hetero-multicomponent metal oxide photocatalysts represent a particularly promising platform for sustainable wastewater remediation.

This review aims to thoroughly analyze the development and application of sandwiched Ti–Cu–Zn nanostructures in advanced oxidation processes (AOP) for agricultural wastewater treatment. It begins with the fundamental principles of photocatalysis and the role of engineered heterojunctions in enhancing charge carrier dynamics and light absorption. The review then provides a comprehensive assessment of synthesis strategies, structural characteristics, and photocatalytic performance across a range of organic and microbial pollutants, with a special focus on reusability and mechanistic insights into ROS generation. In addition, it addresses engineering considerations such as scalability, coating durability, and environmental safety. Finally, the review identifies critical knowledge gaps, particularly in heavy metal remediation, catalyst reusability, and field validation, and offers future perspectives to guide the design and deployment of sustainable photocatalytic systems in real-world agricultural settings.

## 2. Advanced Oxidation Processes in Wastewater Treatment

Advanced oxidation processes (AOPs) are a set of chemical treatment procedures designed to remove organic and inorganic pollutants from wastewater by generating highly reactive radicals, primarily hydroxyl radicals(•OH). These processes are particularly effective in degrading persistent organic pollutants that conventional treatment methods cannot eliminate. AOPs encompass a variety of techniques, including ozonation [27,28], advanced oxidation with Fenton’s reagent [29], photocatalysis [28,30], and nonthermal plasma [31], each with unique mechanisms and applications.

While ozonation and Fenton-based processes often exhibit high degradation efficiencies, they typically rely on continuous chemical inputs, strict pH control, or complex operational requirements, which may limit their long-term sustainability and large-scale deployment. Among these, photocatalytic AOPs stand out because of their ability to harness solar energy, operate under mild conditions, and avoid secondary pollution. For these reasons, this review focuses specifically on photocatalytic AOPs, with an emphasis on hetero-multicomponent metal oxide catalysts as a platform for enhancing charge separation, redox activity, and long-term stability.

**Figure 1:**
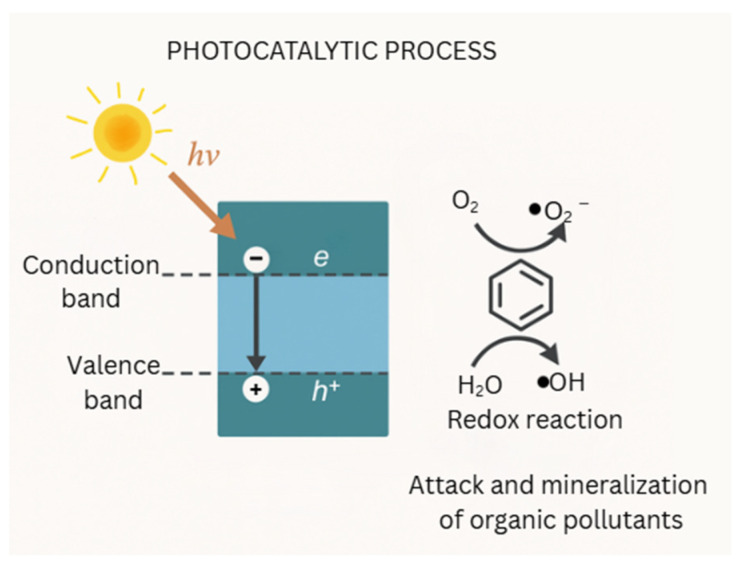
Photocatalysis for pollutant degradation.

In photocatalytic processes, a semiconductor material absorbs photons with energy equal to or greater than its bandgap, resulting in the excitation of electrons (e^−^) into the conduction band and the generation of holes (h^+^) in the valence band. These charge carriers participate in redox reactions that produce ROS, such as •OH and superoxide anion radicals (•O_2_^−^), which attack and mineralize organic pollutants as shown in Figure 1.

Reactive oxygen species (ROS) play a crucial role in the degradation of organic contaminants, primarily through advanced oxidation processes (AOPs). These processes use ROS, such as hydroxyl radicals (•OH) [32,33], superoxide radicals (•O_2_^−^) [34], and peroxides (H_2_O_2_) [32,35] to break down complex organic pollutants into simpler and less harmful compounds as demonstrated in Figure 1. The high reactivity of ROS allows them to modify the chemical structure of organic matter, leading to its degradation or mineralization [36]. This mechanism is particularly effective in various environmental settings, including water, soil, and atmospheric environments [37].

The efficiency of photocatalytic AOPs is strongly influenced by the physicochemical properties of the photocatalyst, including its bandgap, surface area, crystallinity [38], and charge carrier dynamics. AOPs are capable of mineralizing pollutants into harmless end products, making them environmentally benign [39]. They can effectively degrade recalcitrant compounds that are resistant to conventional treatment methods [40]. Hybrid AOPs, which combine different techniques, can improve oxidation capabilities and treatment efficiency [41].

On the other hand, AOPs have high operational costs and energy requirements [42]. Some processes, such as ozonation, may require additional steps to overcome limitations such as mass transfer and reaction rates [27]. The potential formation of toxic by-products requires careful control and optimization [42] of the process. Future research is focused on enhancing the properties of catalysts used in AOPs and developing more efficient hybrid systems. This includes exploring new materials and technologies to reduce costs and improve process efficiency [39]. Combining AOPs with bioremediation and other treatment methods can optimize pollutant removal and reduce operational costs. This integrated approach is gaining traction as a sustainable solution for wastewater treatment [42].

In the context of agricultural wastewater treatment, photocatalytic AOPs offer several advantages. They enable the degradation of low-concentration micropollutants, function effectively in complex water matrices, and are amenable to decentralized, solar-driven systems suitable for rural implementation. However, challenges such as catalyst stability, light penetration, and cost-effective reactor design remain to be addressed. This underscores the need for multifunctional and robust photocatalytic materials like Ti–Cu–Zn sandwiched nanostructures that combine high activity, stability, and environmental compatibility.

## 3. Sandwiched Nanostructures: Design Principles

Sandwiched nanostructures are a fascinating area of research due to their unique properties and potential applications in various fields such as energy storage, catalysis, and electronics. The design principles of these structures involve the strategic layering of different materials to exploit their synergistic effects, enhance performance, and achieve specific functionalities. This approach is evident in the diverse applications and methodologies discussed across the provided papers, which highlight the versatility and innovation in designing sandwiched nanostructures.

### 3.1. Design Principles and Applications

#### 3.1.1. Material Selection and Layering

The choice of materials and their arrangement in a sandwich structure is crucial. For instance, transition metal dichalcogenides sandwiched between graphene, h-BN, and g-C3N4 have been shown to enhance photocatalytic and optoelectronic properties due to their stable structures and expanded band gaps [43]. Similarly, polymer nanocomposites with high dielectric constants and low dielectric losses are achieved by integrating complementary properties of spatially organized multicomponents [44]. Precise synthesis methods are essential for creating stable, high-performance layered sandwich architectures. Atomic layer deposition (ALD) enables nanometer-scale control and defect-free interfaces, as shown in ZnO/TiO_2_ heterostructures and TiO_2_–Cu–ZnO Z-scheme nanocomposites for enhanced photocatalysis [45,46,47]. Layer-by-layer (LbL) assembly allows sequential stacking of materials such as g-C_3_N_4_ and TiO_2_, improving interfacial contact and light harvesting [48,49,50]. Electrophoretic deposition (EPD) offers a scalable route to uniform oxide coatings, demonstrated in TiO_2_ films for antibiotic degradation in greywater [51,52]. Choosing a method aligned with the desired material combination optimizes band alignment, charge separation, and long-term stability.

#### 3.1.2. Synergistic Effects

The interaction between different layers can lead to enhanced properties. For example, sandwich-structured nanocomposites with Ni(OH)_2_ and Poly(methyl methacrylate) Poly(vinylidene fluoride–hexafluoropropylene) (PMMA/P(VDF-HFP)) layers exhibit improved energy storage performance due to the synergistic coupling effects of the layers [53]. In catalysis, MnO_2_–Pd–CeO_2_ hollow spheres demonstrate enhanced stability and activity for CO oxidation, attributed to the strong synergistic effects between Pd and the MnO_2_–CeO_2_ shell [54].

#### 3.1.3. Structural Design and Stability

The structural design of sandwiched nanostructures can significantly impact their stability and performance. The use of hollow sandwich heterostructures, such as those involving transition metal phosphides, provides a unique configuration that combines the advantages of sandwich, hollow, and vertical heterostructures, leading to excellent catalytic performance [55]. Additionally, the incorporation of transition layers in five-layer gradient structures helps reduce dielectric contrast and improve energy storage performance [56].

#### 3.1.4. Biomimetic and Flexible Designs

Inspired by natural structures, biomimetic designs such as honeycomb patterns offer high strength-to-weight ratios, making them suitable for lightweight construction applications [57]. Flexible designs, such as those used in electromagnetic interference shielding materials, leverage the processability and environmental friendliness of materials like carbon fibers and graphene [58].

While sandwiched nanostructures offer numerous advantages, there are challenges and considerations in their design and application. The complexity of self-assembly processes, as seen in DNA origami structures, requires precise control over interaction specificity and structural geometry to ensure efficient folding and assembly [59]. Additionally, achieving a balance between flexibility and structural integrity is crucial, as increased flexibility can introduce variability and potential errors in assembly [59]. These challenges highlight the need for continued research and innovation in the design and fabrication of sandwiched nanostructures to fully realize their potential across various applications.

## 4. Ti–Cu–Zn Nanostructures: Properties and Photocatalytic Behavior

Ti–Cu–Zn nanostructures are promising materials for wastewater treatment due to their enhanced photocatalytic properties. These nanocomposites combine the advantages of individual metal oxides, such as TiO_2_’s high photocatalytic activity, ZnO’s large exciton binding energy, and CuO’s narrow band gap, to create materials with improved efficiency under both UV and visible light irradiation. This section provides a comprehensive overview of their properties, photocatalytic behavior, and synthesis methods.

### 4.1. Properties of Ti–Cu–Zn Nanostructures

#### 4.1.1. Structural and Morphological Properties

The structural and morphological properties of Ti–Cu–Zn nanocomposites are critical in determining their photocatalytic performance. These properties include:

- Crystalline Structure: The nanocomposites typically exhibit a combination of anatase (TiO_2_), wurtzite (ZnO), and monoclinic (CuO) phases, as confirmed by X-ray diffraction (XRD) analysis [60,61,62,63].
- Morphology: Scanning electron microscopy (SEM) and transmission electron microscopy (TEM) reveal quasi-spherical, rod-shaped, or agglomerated morphologies, depending on the synthesis method [61,64,65].
- Surface Area: High specific surface areas, often exceeding 80 m^2^/g, are observed due to the porous nature of the nanocomposites, which enhances photocatalytic activity [61,65].

#### 4.1.2. Optical and Electronic Properties

The optical and electronic properties of Ti–Cu–Zn nanocomposites are tailored to improve photocatalytic efficiency:

- Band Gap: The band gap of these nanocomposites typically ranges between 3.06 and 3.34 eV, allowing absorption in both UV and visible light regions [61,65,66].
- Charge Carrier Separation: The formation of heterojunctions between TiO_2_, ZnO, and CuO suppresses charge-carrier recombination, leading to higher photocatalytic activity [61,63,64].
- Photoluminescence (PL): Lower PL intensity in the nanocomposites indicates reduced exciton recombination, further confirming enhanced charge separation [61,67].

#### 4.1.3. Chemical and Thermal Properties

The chemical and thermal stability of Ti–Cu–Zn nanocomposites is essential for their application in wastewater treatment:

- Thermal Stability: The nanocomposites exhibit stability up to 500 °C, with minimal phase transitions observed during thermal treatment [20,63].
- Chemical Resistance: The materials are resistant to chemical corrosion, making them suitable for repeated use in photocatalytic processes [64,67].

The summary of all properties of the nanostructure is displayed in Table 1.

### 4.2. Photocatalytic Behavior in Wastewater Treatment

#### 4.2.1. Mechanism of Photocatalysis

The photocatalytic activity of Ti–Cu–Zn nanocomposites is driven by the following mechanism:

- Light Absorption: The nanocomposite absorbs light, generating electron–hole pairs.
- Charge Separation: The heterojunctions between TiO_2_, ZnO, and CuO facilitate charge separation, reducing recombination.
- Oxidative Reactions: The holes oxidize water to produce hydroxyl radicals (•OH), while electrons reduce oxygen to form superoxide radicals (•O_2_^−^), which degrade organic pollutants [45,61,64,69].

#### 4.2.2. Degradation Efficiency

The photocatalytic efficiency of Ti–Cu–Zn nanocomposites is superior to that of individual metal oxides:

- Methylene Blue Degradation: Under UV and visible light, the nanocomposites achieve 100% and 98% degradation, respectively, within 2 h [61,62].
- Rhodamine B Degradation: A degradation efficiency of 96.1% is reported for CuO–ZnO nanocomposites under optimized conditions [64].
- Phenol and Azo Dyes: The nanocomposites also exhibit high degradation rates for phenol and azo dyes, with rate constants exceeding 0.04 min^−1^ [60,70,71].
- Antibiotics: Various degradation efficiencies for different antibiotic systems were found to be between 72% and 95% [72,73,74,75].
- Fertilizers: The degradation efficiency of various nitrate and ammonia-based fertilizers using these nanocomposites ranged from 82–92% [76,77,78,79].

#### 4.2.3. Active Species and Stability

The role of reactive oxygen species (ROS) and the stability of the nanocomposites are critical for sustained photocatalytic activity:

- Active Species: Hydroxyl radicals (•OH) are the primary active species responsible for degradation, as confirmed by scavenger experiments [61,69].
- Reusability: The nanocomposites retain up to 75% of their initial activity after four cycles, demonstrating good stability [64,67].

### 4.3. Synthesis Methods

#### Overview of Synthesis Techniques

The synthesis methods of Ti–Cu–Zn nanocomposites involve various methods, each with distinct advantages:

- Sol–Gel Method: This method produces uniform nanocomposites with high surface areas and tailored band gaps [61,62,70].
- Hydrothermal Method: Hydrothermal synthesis allows precise control over particle size and morphology, leading to enhanced photocatalytic activity [60,66,68].
- Biosynthesis: Green synthesis using plant extracts is environmentally friendly and cost-effective, yielding stable nanocomposites [64,67].
- Mechanical Mixing and Wet Impregnation: These methods are simple and cost-effective, suitable for large-scale production [71].
- Spray Pyrolysis: This technique produces thin films with uniform distribution of metal oxides, ideal for solar applications [45,80].
- Gas-Phase Fabrication: This method minimizes liquid waste and produces crystalline nanoparticles with high photocatalytic activity [81].

These methods are summarized with their advantages and disadvantages in Table 2.

### 4.4. Applications in Wastewater Treatment

#### 4.4.1. Target Pollutants

Ti–Cu–Zn nanocomposites are effective against a wide range of pollutants:

- Dyes: Methylene blue, Rhodamine B, and Reactive Orange 16 are efficiently degraded with removal rates between 75 and 90% [61,64,66].
- Phenols: The nanocomposites achieve significant phenol removal under solar irradiation with rates between 54% and 80% [71].
- Azo Dyes: High degradation efficiency for azo dyes is reported, with rate constants exceeding 0.04 min^−1^ [70].
- Antibiotics: Ciprofloxacin, tetracycline, sulfamethoxazole, and chlortetracycline hydrochloride are efficiently degraded with removal rates between 72–100% under visible and UV light using these nanocomposites [82,83,84].
- Pesticides: Imidacloprid is completely mineralized (~100%) under solar-simulated light with efficiency above 91% after multiple reuse cycles [85].
- Herbicides: 2,4-dichlorophenol (2,4-DCP) and related chlorophenols achieve ~92% TOC mineralization using nanostructures under visible light [86].
- Fertilizers (nitrate, ammonia nitrogen): Photocatalytic systems have demonstrated notable performance in fertilizer-related wastewater treatment. Complete nitrate removal with approximately 70% selective conversion to ammonium under UV light [87], while 61% of ammonia-nitrogen was degraded within 3 h, retaining 52% efficiency after three reuse cycles [88].

#### 4.4.2. Antibacterial Activity

In addition to pollutant degradation, the nanocomposites exhibit antibacterial properties:

- Bacterial Strains: The materials are effective against Escherichia coli and Staphylococcus aureus, with over 99% colony degradation [65,68].
- Mechanism: The generation of ROS and direct contact with bacterial cells contribute to their antibacterial activity [65,68].

#### 4.4.3. Comparative Photocatalytic Performance of TiO_2_-Based Systems Against Key Contaminants

To evaluate the practical relevance of TiO_2_-based photocatalysts, it is important to compare their efficiency against various environmental contaminants under different light conditions. Integration of CuO and ZnO into TiO_2_ frameworks has consistently demonstrated improved photocatalytic performance, especially under visible and solar irradiation. These ternary systems exhibit faster degradation rates and a wider compatibility of pollutants compared to single- or bimetallic configurations. Table 3 summarizes the photocatalytic efficiencies of representative TiO_2_-based systems against common pollutants such as methylene blue, heavy metals, and bacteria, highlighting the synergistic effect of sandwiched nanostructures. It should be noted that reported catalytic performances across studies are not directly comparable due to variations in light source intensity and spectrum, catalyst dosage, pollutant concentration, solution chemistry, and reactor configuration. Consequently, degradation efficiencies and rate constants reported herein should be interpreted as indicative trends rather than absolute benchmarks of catalyst superiority.

Current evidence for heavy metal remediation using Ti–Cu–Zn-based catalysts remains limited, and most available studies focus on adsorption or indirect redox interactions rather than systematic photocatalytic removal. Therefore, discussions in this section clearly distinguish experimentally demonstrated observations from proposed research opportunities. While multicomponent oxide architectures offer theoretical advantages for heavy metal immobilization and redox transformation, their practical performance remains largely unvalidated and represents an important direction for future investigation.

Ti–Cu–Zn nanostructures are versatile materials with enhanced photocatalytic properties, making them ideal for wastewater treatment. Their high surface area, suppressed charge-carrier recombination, and tailored band gaps enable efficient degradation of organic pollutants under both UV and visible light. Various synthesis methods, including sol–gel, hydrothermal, and biosynthesis, offer flexibility in tailoring their properties. These nanocomposites also exhibit antibacterial activity, further expanding their applications. Future research should focus on scaling up synthesis methods and improving stability for real-world applications.

## 5. Mechanistic Insights into Photocatalytic AOPs

Understanding the mechanisms underlying the enhanced photocatalytic activity of Ti–Cu–Zn nanostructures is key to optimizing their design and application. The synergistic interaction among titanium, copper, and zinc plays a crucial role in the promotion of interfacial charge transfer and reactive oxygen species (ROS) generation, which directly influences pollutant degradation pathways.

**Figure 2:**
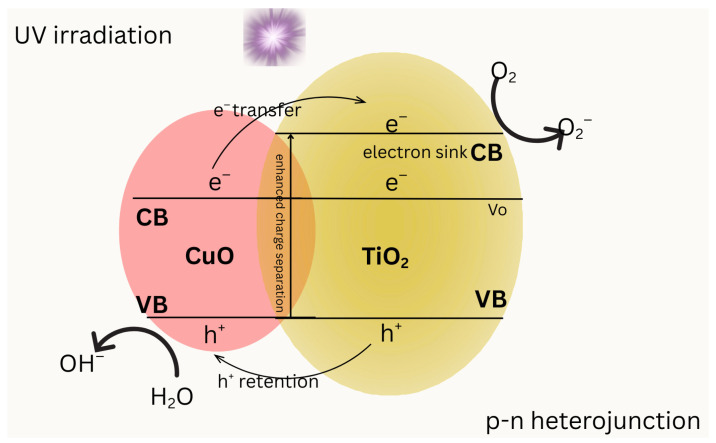
Schematic illustration of charge transfer and photocatalytic reaction pathways in a TiO_2_–CuO heterojunction under UV irradiation.

The integration of copper (Cu) into titanium dioxide (TiO_2_) photocatalysts has been extensively studied to enhance the efficiency of photocatalytic advanced oxidation processes (AOPs). The mechanistic insights into these processes in Figure 2 reveal that Cu plays a significant role in modifying the electronic properties [97] and catalytic activity of TiO_2_ [98], thus improving its performance in various photocatalytic applications. The interaction between Cu and TiO_2_ can lead to enhanced adsorption [98,99], improved charge separation [97] and increased visible light activity, which are essential for effective photocatalysis. Upon excitation, photogenerated electrons migrate from the CuO conduction band to the TiO_2_ conduction band, where TiO_2_ acts as an electron sink, facilitating the reduction in dissolved O_2_ to superoxide radicals (O_2_•^−^). Simultaneously, photogenerated holes are preferentially retained in the CuO valence band, promoting the oxidation of surface-adsorbed H_2_O/OH^−^ to hydroxyl radicals (•OH). The interfacial junction between TiO_2_ and CuO enhances charge separation and suppresses electron–hole recombination. Band positions are schematic and shown to illustrate relative alignment rather than absolute energetic values.

**Figure 3:**
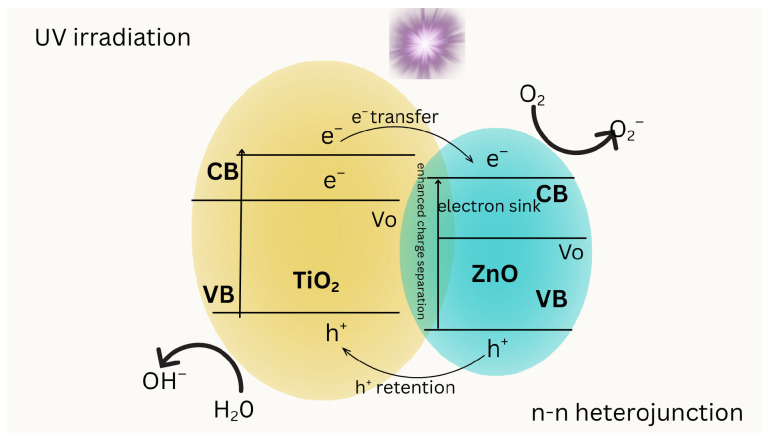
Schematic representation of the photocatalytic mechanism in a TiO_2_–ZnO heterojunction.

Integration of zinc into TiO_2_ structures in Figure 3, through doping or the formation of heterojunctions, significantly influences photocatalytic activity by altering electronic properties and facilitating the charge dynamics [46]. Zn doping in TiO_2_ creates oxygen vacancies, which are essential to improve photocatalytic activity. These vacancies act as electron traps to reduce the rates of electron recombination and increase the oxidation potential of the valence band [47]. ZnO improves light harvesting by extending absorption to visible wavelengths and reducing carrier recombination through decreased surface defects and improved charge separation [100]. Under UV illumination, electrons excited in the TiO_2_ conduction band transfer to the ZnO conduction band, where ZnO functions as an electron sink and promotes O_2_ reduction to O_2_•^−^ species. Concurrently, photogenerated holes remain in the TiO_2_ valence band, enabling the formation of hydroxyl radicals (•OH) from adsorbed water or hydroxide ions. The TiO_2_–ZnO interface facilitates enhanced charge separation, reducing recombination losses and improving photocatalytic efficiency. Band positions are depicted schematically for mechanistic clarity.

**Figure 4:**
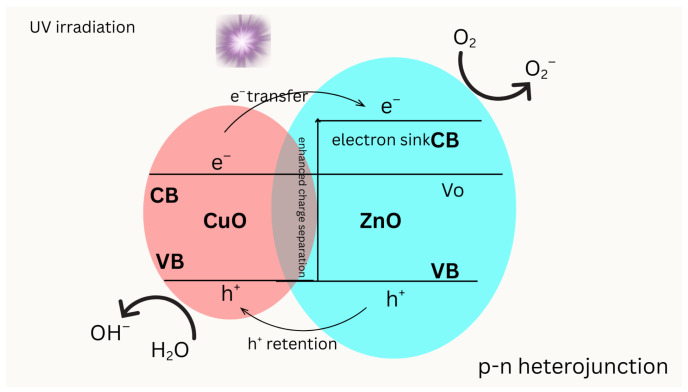
Charge transfer mechanism in a CuO–ZnO heterojunction under photocatalytic conditions.

Copper-zinc (Cu-Zn) photocatalytic systems leverage the synergistic effects of Cu and Zn to improve light absorption, charge separation, and catalytic efficiency. The mechanistic insights into these systems in Figure 4 reveal the critical roles of heterojunction formation, electron–hole pair dynamics, and radical generation in the driving of photocatalytic reactions. Cu doping in ZnO alters the electronic structure, enhancing visible light absorption and creating localized electronic states that facilitate charge transfer [101]. In Cu-Zn systems, the interaction between Cu single atoms and ZnS can regulate electronic structures, optimizing the adsorption of intermediates and promoting electron transfer, which is essential for processes such as hydrogen evolution [102]. The ecological impact of these systems is positive, as degradation intermediates generally exhibit reduced toxicity, thereby minimizing environmental risks during the photocatalytic process [103]. The stability of these photocatalysts is further evidenced by their consistent performance over repeated use, as seen in CuO–ZnO systems, which maintain high degradation rates over several cycles [104]. Photoexcited electrons migrate from the CuO conduction band to the ZnO conduction band, where ZnO serves as the primary electron sink and supports superoxide radical (O_2_•^−^) generation. Meanwhile, holes are retained in the CuO valence band, contributing to oxidative pathways such as hydroxyl radical (•OH) formation. The CuO–ZnO interfacial junction promotes spatial separation of charge carriers, thereby suppressing recombination. Band alignment is schematic and intended to illustrate relative energetic trends.

**Figure 5:**
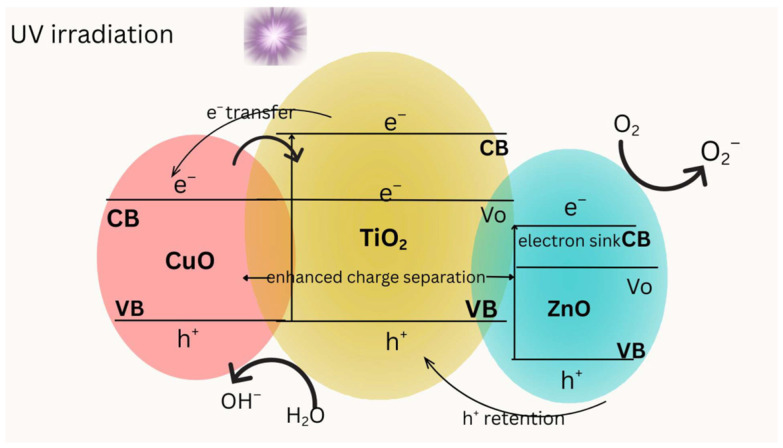
Schematic illustration of charge transfer and reactive oxygen species (ROS) generation in a Ti–Cu–Zn ternary heterojunction under UV irradiation.

The combined effect is a heterojunction system in which band bending and Schottky junctions form at the Ti–Cu and Ti–Zn interfaces, promoting directional charge migration. These interfacial electric fields improve the spatial separation of h^+^ and e^−^, ensuring longer-lived ROS as seen in Figure 5. The process can be summarized in the following mechanistic pathway [45,61]:

1. TiO_2_ absorbs light → e^−^ + h^+^ generation
2. e^−^ transfer to Cu^2+^ → Cu^+^ and •O_2_^−^
3. h^+^ reacts with H_2_O/OH^−^ → •OH
•O_2_^−^ and •OH and degrade pollutants
5. The surface sites of Zn^2+^ stabilize charges and inhibit recombination

Upon excitation, photogenerated electrons preferentially accumulate in the ZnO conduction band, which acts as the dominant electron sink and facilitates O_2_ reduction to O_2_•^−^. Photogenerated holes are primarily retained in the TiO_2_ valence band, with additional hole trapping in CuO, enabling efficient •OH formation. Oxygen vacancy (Vo) states at the interfaces further enhance charge separation and inhibit recombination. The dual heterointerfaces between CuO–TiO_2_ and TiO_2_–ZnO collectively promote enhanced charge separation and improved photocatalytic performance. Band positions are schematic and shown for relative alignment rather than absolute energetic values. This cooperative interaction improves degradation efficiency, broadens light absorption, and ensures sustained catalytic performance in real wastewater matrices.

### Strengths and Limitations of Ti–Cu–Zn Systems Compared to Single/Bimetallic Systems

Although numerous studies have explored the photocatalytic performance of single and binary metal oxide systems, the emergence of ternary Ti–Cu–Zn nanostructures offers a promising advancement in both degradation efficiency and functional versatility. These systems benefit from broadened light absorption, enhanced charge separation, and dual capacity for pollutant degradation and antimicrobial action. However, challenges such as metal-ion leaching, scalability, and long-term environmental safety persist. Table 4 provides a comparative overview of the strengths and limitations of TiO_2_-based systems, highlighting the unique advantages and trade-offs of the sandwiched Ti–Cu–Zn configuration compared to traditional single and bimetallic alternatives.

## 6. Stability, Reusability, and Environmental Safety

One of the primary challenges in the practical application of nanomaterials in wastewater treatment is ensuring long-term catalyst stability and reusability without compromising environmental safety. For sandwiched Ti–Cu–Zn nanostructures to be effectively deployed in real-world systems, their mechanical adhesion, resistance to photo corrosion, and potential for heavy metal leaching must be critically evaluated. The durability, reusability, and environmental safety of coatings on steel substrates are critical factors in extending the lifespan and functionality of steel in various applications. Coatings must withstand harsh environmental conditions, resist corrosion, and maintain their protective properties over time. Recent research has focused on developing advanced coatings that meet these criteria, using innovative materials and methods to enhance performance [112,113].

### 6.1. Coating Durability on Steel Substrates

When applied to solid supports such as AISI 316 stainless steel, Ti–Cu–Zn nanomaterials require robust immobilization strategies [114,115]. Spray coating [116,117] and electrophoretic deposition (EPD) [28,118,119] have been used to adhere the photocatalyst to the substrates, minimizing particle loss during operation. Among these, spray coating provides better scalability but may suffer from poor interfacial adhesion if it is not optimized. The incorporation of methanol or other organic solvents during the dispersion step has been shown to enhance coating uniformity and surface anchoring. Mechanical abrasion tests [120] and water-jet exposure experiments [121,122] can be used to simulate real flow conditions and validate the durability of the coating during repeated use cycles. These tests are crucial for assessing the performance of coatings in environments where they are subjected to erosive forces, such as in hydraulic structures, aircraft, and marine applications, even though they are not without limitations.

### 6.2. Photo-Corrosion Resistance and Structural Integrity

TiO_2_-based catalysts are generally stable under UV and visible irradiation. However, the introduction of Cu and Zn can introduce structural vulnerabilities if not properly integrated [123]. For example, Zn-doped TiO_2_ coatings have shown improved corrosion resistance, but under light irradiation, increased electrical conductivity can lead to reduced corrosion resistance due to excessive charge transfer [124]. Stabilizing these ions within a well-defined crystalline or amorphous matrix, or through a heterojunction design, can mitigate degradation. Posttreatment sintering [125] or the formation of protective oxide layers [126,127] can also improve the long-term photochemical stability of reactive sites and enhance the structural integrity of the materials.

### 6.3. Catalyst Reusability

Effective photocatalysts must maintain high degradation efficiency over multiple treatment cycles. Several studies have shown that multicomponent nanostructures can retain more than 80–90% of their initial activity after 5–10 cycles of antibiotic or dye degradation [128]. Performance losses are typically due to surface fouling [129], partial leaching of active sites [130], or phase transformation [131]. Regeneration strategies such as functionalizing nanostructures with stabilizing agents or coatings [132], optimizing the morphology and surface chemistry [133], or implementing effective recycling and regeneration processes, such as magnetic recovery or surface cleaning [129,130] can help restore activity.

### 6.4. Leaching Behavior and Ecotoxicological Impact

The potential release of metal ions, particularly Cu^2+^ and Zn^2+^, into treated water must be carefully controlled to prevent secondary pollution [134,135]. In several studies, measured Cu^2+^ concentrations approach or exceed guideline limits for aquatic environments, highlighting the necessity of rigorous leaching assessments. Inductively coupled plasma mass spectrometry (ICP-MS) is commonly used to quantify leaching levels after each treatment cycle [136]. In well-engineered systems, leaching is often found to be below regulatory limits (<0.1 mg/L) depending on pH, irradiation time, and catalyst synthesis route. However, environmental risk assessment through bioassays (e.g., Daphnia magna, algal growth inhibition tests) should accompany performance evaluations [137,138] to ensure the safe deployment of such catalysts in agricultural contexts. Also, testing methodologies vary widely, including batch immersion tests, photocatalytic cycling experiments, and post-reaction Inductively Coupled Plasma–Optical Emission Spectroscopy (ICP-OES) analysis, complicating cross-study comparison. Establishing standardized leaching protocols and correlating metal release with catalyst structure and operational conditions remains a critical requirement for safe-by-design catalyst development.

Ultimately, achieving a balance between high photocatalytic performance and environmental safety will determine the success of Ti–Cu–Zn nanostructures in decentralized wastewater treatment systems. The development of smart coatings, eco-friendly synthesis routes, and comprehensive life-cycle assessments will be critical moving forward.

## 7. Engineering Considerations and Scale-Up Potential

Despite significant laboratory-scale successes, the transition of multicomponent nanostructures from bench-scale prototypes to full-scale wastewater treatment systems requires careful engineering consideration. This section outlines key factors influencing scale-up feasibility, including system integration, material cost, reactor design, and performance under real wastewater matrices. Translating batch photocatalysis to continuous water processes requires immobilization and reactor engineering. Suitable strategies include ALD/EPD coatings on porous supports, Layer-by-Layer (LbL) films on polymeric membranes, and integration into photoelectrochemical flow cells. Each route balances trade-offs: immobilized systems ease catalyst recovery but may reduce mass transfer; photocatalytic membranes offer simultaneous separation and degradation but increase fouling risk. We summarize practical recommendations and reported engineering metrics below.

### 7.1. Integration into Decentralized Treatment Units

The application of sandwiched nanostructures is well-suited to decentralized wastewater systems, particularly in rural or agricultural settings lacking centralized treatment infrastructure. The integration of nanostructures into treatment units involves both top-down and bottom-up approaches. Their ability to operate under solar or low-power light sources makes them attractive for off-grid applications. Immobilization of the nanomaterials on substrates such as stainless-steel mesh or glass plates facilitates integration into fixed-bed or flow-through photoreactors. Modular design and low maintenance requirements are also desirable features for rural deployment. These methods must be adapted to ensure compatibility with existing microelectronic and semiconductor technologies, which are essential for the development of smart treatment systems [139].

### 7.2. Reactor Design and Light Penetration Efficiency

Efficient photocatalytic operation requires maximizing photon absorption and pollutant-catalyst contact [140]. Flat-plate, annular, or compound parabolic concentrator (CPC)-based reactors can be engineered to ensure uniform irradiation. Different reactor types, such as fixed-bed, slurry, and structured reactors, offer unique advantages and challenges for scaling up nanostructure production. Structured reactors, which involve depositing nanoparticles on solid supports, are particularly promising for continuous operations and large-scale applications [141]. The placement and orientation of Ti–Cu–Zn-coated surfaces should minimize shadowing effects and enhance light distribution. Coupling UV–Vis or solar light with internal reflectors can further improve photocatalytic efficiency in large volumes. The design of nanostructures can enhance light scattering and absorption, improving catalytic efficiency. The unique topological features of these structures reduce charge migration distances, enhancing light harvesting in confined spaces [142].

### 7.3. Cost and Scalability of Synthesis

A systems materials science approach is essential for designing nanocomposites with hierarchical structures that can be scaled up without losing performance. This involves understanding and manipulating interactions across multiple length scales, from molecular to macroscopic levels, to achieve desired properties [143]. While sol–gel, hydrothermal, and spray-coating methods are promising, they must be optimized for cost, reproducibility, and throughput. Ensuring reproducible production of nanostructures is a significant challenge in scale-up. The implementation of nano-CMC (chemistry, manufacturing, and controls) strategies, including stability checks and analytical methods, is essential for maintaining quality and meeting regulatory specifications [144]. Methanol-assisted dispersion or templating steps must be compatible with large-batch or roll-to-roll manufacturing. Additionally, sourcing and stabilizing Cu and Zn precursors economically without sacrificing material performance is key. Life-cycle analysis (LCA) and techno-economic assessment (TEA) should be employed to evaluate process sustainability.

### 7.4. Performance in Complex Wastewater Matrices

Agricultural runoff often contains a mixture of antibiotics, fertilizers, organic matter, and suspended solids. These matrix components may interfere with ROS generation, block active sites, or reduce light transmittance. Pre-treatment steps such as filtration or sedimentation may be required before AOP application. Combining nanomaterials with conventional treatment technologies, such as activated sludge processes and membrane bioreactors, can enhance overall treatment efficiency and facilitate scale-up. This integration can help overcome limitations of traditional methods and improve pollutant removal from complex wastewater matrices [145]. Surface modification of nanostructured adsorbents enhances their affinity for specific pollutants, which is crucial for effective wastewater treatment. This involves tailoring the surface properties to target contaminants like heavy metals and organic compounds commonly found in agricultural runoff [146]. Pilot-scale studies must therefore assess catalyst robustness in simulated and actual wastewater streams.

### 7.5. Automation, Monitoring, and Maintenance

To enable long-term and remote operation, sandwiched nanostructure-based systems can be equipped with basic automation components such as light intensity sensors [147], flow controllers, and photocatalyst fouling indicators. Real-time monitoring of effluent quality and predictive maintenance tools could reduce downtime and ensure consistent pollutant removal performance [147]. The deployment of sensor-driven solutions in wastewater treatment facilities requires a workforce with specialized skills in both environmental engineering and data analysis. Bridging the gap between current training programs and the skills needed for these advanced technologies is essential. Strengthening partnerships for data sharing and field testing of new technologies can also accelerate innovation and make it more accessible to smaller facilities [147].

### 7.6. Sustainability Considerations

Ensuring the sustainability of nanostructure-based treatment units is critical. This involves evaluating the environmental impact of nanomaterials throughout their lifecycle, from production to disposal, and developing frameworks to guide sustainable design and implementation [148].

### 7.7. Commercialization and Market Potential

The commercialization of nanostructure-based water treatment technologies is gaining traction, with applications ranging from industrial wastewater treatment to decentralized drinking water systems. The development of nanocomposites and advanced membranes is particularly promising for addressing global water challenges [149].

### 7.8. Regulatory & Deployment Checklist for Agricultural Use

To ensure the practical translation of Ti–Cu–Zn nanostructures from laboratory-scale photocatalysts to field-level wastewater treatment systems, it is essential to align their deployment with environmental safety standards and regulatory expectations. While laboratory results highlight strong degradation and antibacterial efficiencies, long-term environmental behavior, such as potential leaching of Cu^2+^ and Zn^2+^, remains insufficiently assessed. Additionally, data on catalyst reusability, field scalability, and compatibility with real agricultural runoff are scarce. Table 5 presents a regulatory and deployment checklist that outlines the critical parameters for safe and effective integration of sandwiched nanostructures into agricultural wastewater treatment systems.

Taken together, these engineering considerations highlight the importance of an interdisciplinary approach combining materials science, environmental engineering, and systems integration. Pilot demonstrations and stakeholder engagement will be important next steps in translating Ti–Cu–Zn photocatalytic systems from the laboratory to practical wastewater solutions.

## 8. Future Perspectives and Research Opportunities

Despite significant progress in multicomponent nanostructures for photocatalytic wastewater treatment, several opportunities remain for future investigation. Key directions include:

- Tailored band engineering using advanced dopants or quantum heterostructures to expand light absorption into the near-infrared region and enhance charge carrier lifetimes.
- Hybrid integration of AOP, such as coupling heterocatalysts with ozonation, Fenton, or electrochemical oxidation, to boost degradation efficiency.
- Real-time contaminant monitoring technologies combined with intelligent photocatalyst systems for adaptive water treatment.
- Green and scalable synthesis methods that eliminate toxic solvents, reduce energy input, and enable on-site fabrication.
- Ecotoxicity and life cycle assessment (LCA) studies to ensure safe deployment in agricultural, municipal, and industrial settings.
- Furthermore, fundamental studies on catalyst-pollutant interaction kinetics and reactive intermediate tracking will be vital for mechanism elucidation and rational catalyst design.

Recent studies increasingly demonstrate that catalyst performance in wastewater treatment is highly sensitive to experimental configuration, making direct comparison across reports non-trivial. For instance, recent investigations on multicomponent and hybrid oxide catalysts have reported enhanced degradation efficiencies under visible-light irradiation; however, these outcomes are often achieved under markedly different light sources, catalyst loadings, and pollutant concentrations. Studies published by Ma et al. [150] highlight that apparent performance gains may originate from optimized reactor geometries or synergistic adsorption–photocatalysis coupling rather than intrinsic catalytic superiority. Similarly, other articles emphasize that while advanced oxide catalysts can exhibit high short-term removal efficiencies, long-term stability and metal leaching remain critical bottlenecks. These findings collectively underscore the need for standardized benchmarking protocols and caution against over-interpreting isolated degradation efficiencies when evaluating emerging hetero-multicomponent systems.

### Knowledge Gaps and Future Perspectives

Despite promising results, several gaps must be addressed to unlock the full potential of multicomponent nanostructures:

- Need for in situ characterization techniques: Current studies often rely on post-reaction analyses. Incorporating in situ techniques (e.g., X-ray Photoelectron Spectroscopy (XPS), Diffuse Reflectance Infrared Fourier Transform Spectroscopy (DRIFTS), and Electron Paramagnetic Resonance (EPR) under operational conditions) would help track the dynamics of charge transfer, surface species, and ROS generation.
- Reusability and stability studies: Current studies have no experiments on reusability and stability, and therefore, new studies going forward have to focus on this. Ensuring that the synthesized catalyst can achieve nothing less than 15% degeneration after 5 runs will be a huge win for the system, as this is the minimal requirement seen in other single or binary catalyst systems.
- Heavy metal reduction: Industrial, pharmaceutical, and agricultural wastewater systems tend to contain heavy metals such as chromium, cadmium, lead, etc. More studies must be carried out on the efficiency of removing these heavy metals from wastewater, as current studies do not have citable references for these elements.
- Exploring new dopants and structural motifs: Beyond Cu and Zn, incorporation of rare earth, non-metal (e.g., N, S), or perovskite-inspired interfaces may further tune band alignment and enhance activity.
- Policy and regulatory alignment: For field deployment, regulatory clarity is required regarding nanoparticle release, reuse standards, and permissible leaching thresholds. Engagement with environmental authorities, farmers, and policymakers is essential to facilitate technology transfer.

Addressing these gaps will not only improve photocatalyst functionality but also support regulatory approvals and public trust.

## 9. Conclusions

Hetero-multicomponent metal oxide catalysts represent an emerging and versatile platform for addressing the complexity of modern wastewater streams. This review has shown that Ti–Cu–Zn-based systems, through synergistic band alignment, multi-redox activity, and enhanced interfacial charge transfer, can outperform conventional single-component and binary catalysts across a range of photocatalytic, Fenton-like, and adsorption-assisted processes.

While substantial progress has been achieved in degrading organic micropollutants, this review highlights persistent challenges related to performance benchmarking, catalyst stability, metal leaching, and scalability. In particular, heavy metal remediation using multicomponent oxide systems remains largely unexplored and warrants systematic investigation.

Future research should prioritize rational heterostructure design, standardized testing protocols, quantitative assessment of leaching and ecotoxicological risks, and the integration of these catalysts into decentralized and nature-based treatment systems. By addressing these challenges, hetero-multicomponent catalysts—especially Ti–Cu–Zn architectures—are well positioned to play a meaningful role in sustainable and economically viable wastewater treatment technologies.

## Figures and Tables

**Table 1:** Physicochemical Properties of Ti–Cu–Zn Nanostructures.

Property	Typical Range	Functionality	References
Band gap	2.2–3.3 eV	Controls light absorption	Taufik et al., 2017 [61]
Surface area	60–120 m^2^/g	More active sites for degradation	Taufik et al., 2017 [61]
Crystalline phases	Anatase (TiO_2_), Monoclinic (CuO), Wurtzite (ZnO)	Phase synergy	Kumar et al., 2017 [68]
Morphology	Spherical, rod-shaped, agglomerated	Affects light harvesting	Yitagesu et al., 2024 [67]

**Table 2:** Synthesis Methods for Ti–Cu–Zn Nanocomposites.

Synthesis Method	Description	Pros	Cons	References
Sol–Gel Method	Produces uniform nanocomposites with high surface areas and tailored band gaps.	Simple equipment, fine compositional control, homogeneous mixing of precursors.	Long processing times, potential for cracking during drying/calcination.	Taufik et al., 2017 [61] Albert et al., 2015 [62] Baig et al., 2024 [70]
Hydrothermal Method	Uses high-temperature, high-pressure aqueous conditions to control particle size and morphology.	Excellent control over crystal growth, high purity, and energy efficiency for certain systems.	Requires an autoclave, limited scalability for industrial production.	Malakootian et al., 2021 [60] Nassar et al., 2024 [66] Kumar et al., 2017 [68]
Biosynthesis	Employs plant extracts as reducing/stabilizing agents for green synthesis.	Eco-friendly, cost-effective, low-toxicity byproducts.	Batch-to-batch variability, limited control over particle size/shape.	Jeevarathinam et al., 2024 [64] Yitagesu et al., 2024 [67]
Mechanical Mixing and Wet Impregnation	Physically mixes powders or impregnates supports with precursors, followed by drying/calcination.	Low-cost, scalable, straightforward process.	Non-uniform distribution of active sites, possible agglomeration.	Mohammadi et al., 2016 [71]
Spray Pyrolysis	Aerosolized precursor solution is thermally decomposed to form thin films or powders.	Uniform film deposition, good for large-area coatings, compatible with dopants.	Requires specialized spray equipment and higher energy use.	Chen et al., 2022 [45] Mrabet et al., 2023 [80]
Gas-Phase Fabrication	Vapor-phase routes produce crystalline nanoparticles with minimal liquid waste.	High purity, excellent crystallinity, and a clean process.	High cost, requires advanced equipment and expertise.	Hudandini et al., 2024 [81]

**Table 3:** Photocatalytic Performance of TiO_2_-based Systems Against Key Contaminants.

Pollutant	Catalyst System	Light Source	Degradation Efficiency (%)	Time (min)
Methylene Blue	TiO_2_ [89]	UV	62.72	120
	TiO_2_–CuO [90]	UV	92.31	120
	TiO_2_–ZnO [91]	UV	96.4	720
	Ti–Cu–Zn [61]	UV	100	120
Heavy metal	TiO_2_ [92]	Visible	88	120
	TiO_2_–CuO [93]	Visible	95	45
	TiO_2_–ZnO [94]	Visible	53	288
	Ti–Cu–Zn	-	-	-
Bacteria	TiO_2_ [28]	UV	100	10
	TiO_2_–CuO [95]	Visible	98	60
	TiO_2_–ZnO [96]	UV	98	30
	Ti–Cu–Zn [68]	Visible	99	30–120

**Table 4:** Comparison of TiO_2_-based systems.

System	Strengths	Limitations	References
TiO_2_ only	Highly stable, inexpensive, well-known, safe in most environments	UV-only activity, high recombination, limited ROS production	Ghamarpoor et al., 2024 [105]
TiO_2_–CuO	Enhanced visible-light activity, better charge separation, Schottky junction promote electron migration.	Cu^2+^ leaching risk; CuO instability in repeated cycles; poor scalability	Shi et al., 2019 [106], A’srai et al., 2023 [107]
TiO_2_–ZnO	Stronger ROS production; antibacterial synergy; better charge migration	Wide band gap (3.3 eV); ZnO is prone to photo corrosion in aqueous systems	Ghamarpoor et al., 2024 [105], Kubiak et al., 2019 [108]
Ti–Cu–Zn	Combined broad-spectrum light response; superior ROS; dual action (degradation + antibacterial); lower e^−^/h^+^ recombination	Still under development; leaching of Cu/Zn and durability under real water conditions remain concerns	Malakootian et al., 2021 [60], Taufik et al., 2017 [61], Mohammadi et al., 2016 [71], Abdelfattah and El-Shamy, 2024 [109]
Environmental Safety: Ti–Cu–Zn	Effective pollutant degradation with reduced chemical inputs; no need for chemical oxidants; low toxicity at moderate loading	Requires leachate testing and chronic toxicity assessments (Cu/Zn risk); reusability beyond 5 cycles is underexplored	Abdelfattah and El-Shamy., 2024 [109], Joonas et al., 2019 [110], Azizi-lalabadi et al., 2019 [111]

**Table 5:** Regulatory & Deployment Checklist for Agricultural Use.

Criteria	Current Status	Recommendation
Leaching Safety (Cu/Zn)	Partial data	Add bioassays (e.g., *Daphnia*, algal tests)
Catalyst Reuse Data	<5 cycles reported	Target ≥10 with <15% performance loss
Field Testing	Rare	Needed in realistic Agri-runoff systems
Policy Guidelines	Lacking	Propose collaboration with local agencies

## Data Availability

No new data were created or analyzed in this study. Data sharing is not applicable to this article.
